# Serum α1-AT Levels and *SERPINA1* Molecular Analysis in Breast Cancer: An Experimental and Computational Study

**DOI:** 10.3390/diseases13010001

**Published:** 2024-12-24

**Authors:** Guadalupe Ávalos-Navarro, Luis A. Bautista-Herrera, Asbiel Felipe Garibaldi-Ríos, Ramiro Ramírez-Patiño, Marisol Gutiérrez-García, Perla Briseño-Álvarez, Luis Felipe Jave-Suárez, Emmanuel Reyes-Uribe, Martha Patricia Gallegos-Arreola

**Affiliations:** 1 Departamento de Ciencias Médicas y de la Vida, Centro Universitario de la Ciénega (CUCIÉNEGA), Universidad de Guadalajara, Av. Universidad 1115, Lindavista, Ocotlán 47820, Jalisco, Mexico; guadalupe.avalos5337@academicos.udg.mx (G.Á.-N.); ramiro.ramirez@academicos.udg.mx (R.R.-P.); emmanuel.reyes@academicos.udg.mx (E.R.-U.); 2 Departamento de Farmacobiología, Centro Universitario de Ciencias Exactas e Ingenierías (CUCEI), Universidad de Guadalajara, Blvd. Marcelino García Barragán 1421, Olímpica, Guadalajara 44430, Jalisco, Mexico; luis.bautista4106@academicos.udg.mx; 3 Doctorado en Genética Humana, Centro Universitario de Ciencias de la Salud (CUCS), Universidad de Guadalajara (UdeG), Guadalajara 44340, Jalisco, Mexico; asbiel.garibaldi4757@alumnos.udg.mx; 4 División de Genética, Centro de Investigación Biomédica de Occidente (CIBO), Instituto Mexicano del Seguro Social (IMSS), Sierra Mojada 800, Independencia Oriente, Guadalajara 44340, Jalisco, Mexico; 5 Licenciatura en Químico Farmacéutico Biólogo, Centro Universitario de Ciencias Exactas e Ingenierías (CUCEI), Universidad de Guadalajara, Blvd. Marcelino García Barragán 1421, Olímpica, Guadalajara 44430, Jalisco, Mexico; marisol.gutierrez.qfb@gmail.com; 6 Licenciatura en Químico Farmacéutico Biólogo, Centro Universitario de la Ciénega (CUCIÉNEGA), Universidad de Guadalajara, Av. Universidad 1115, Lindavista, Ocotlán 47820, Jalisco, Mexico; patricia.briseno@alumnos.udg.mx; 7 División de Inmunología, Centro de Investigación Biomédica de Occidente (CIBO), Instituto Mexicano del Seguro Social (IMSS), Guadalajara 44340, Jalisco, Mexico; lfjave@gmail.com

**Keywords:** breast cancer, molecular subtype, clinical stage, soluble levels, alpha1 antitrypsin, *SERPINA1*

## Abstract

Background/Objectives: Breast cancer (BC) is a heterogeneous disease with multifactorial origins, including environmental, genetic, and immunological factors. Inflammatory cytokines, such as alpha 1 antitrypsin (α1-AT), are increased in BC and affect physiological and pathological conditions. This study aimed to evaluate the serum levels of α1-AT and perform a computational analysis of *SERPINA1* in BC, as well as their association with molecular subtypes and clinical features. Methods: For the experimental analysis, we evaluated 255 women with BC and 53 healthy women (HW) in a cross-sectional study. Molecular subtypes were identified by immunohistochemistry and TNM was used for clinical staging. Soluble levels of α1-AT were quantified by ELISA. Computational analysis of *SERPINA1* expression was performed using GEPIA and cBioPortal. Results: α1-AT was increased in BC women versus HW (75.8 ng/mL vs. 532.2 ng/mL). Luminal A had higher concentration (547.5 ng/mL) than Triple Negative (TN) (484.1 ng/mL), but the levels were not associated with clinical stage. The computational analysis showed that *SERPINA1* is overexpressed in BC with differential expression among subtypes; its overexpression is associated with a better prognosis, longer disease-free survival, and overall survival. Conclusions: α1-AT levels are increased in women with BC women compared to HW. The Luminal A subtype shows higher soluble protein levels than the TN one. Furthermore, *SERPINA1* mRNA overexpression in BC is linked to a protective effect.

## 1. Introduction

Breast cancer (BC) is a heterogeneous disease of a multifactorial origin, in which genetic and environmental factors are involved [1], as well as immunologic factors, where proinflammatory cytokines are highlighted [2]. BC is the most common cancer affecting women [3] and cause of death for cancer in women around the world, according to data from the World Health Organization (WHO). To detect BC, the methods used are mammography and self-examination, which assist through early diagnosis and treatment to reduce mortality [4]. The biomarkers defined by immunohistochemistry (IHC) for the diagnostic pathology of the breast [5] are the positive hormonal receptors, estrogen and the progesterone receptor (ER and PR, respectively), as well as, receptor 2 of human epidermal growth factor (HER2) and Ki67 cell proliferation index, which have been very useful to define the subrogated molecular phenotypes that guide with the aggressiveness of the tumor and treatment. The molecular subtypes are classified as Luminal A, Luminal B, HER2, and TN [2,5,6,7,8,9,10]. With respect to the clinical stage, it depends upon the size and type of tumor, whereas stage 0 describes non-invasive tumors, and advanced stage 4 describes invasive tumors [5,10].

In cancer, different proteins are involved in the microenvironment, such as alpha 1 antitrypsin (α1-AT), a member of the serine protease inhibitor (serpins) family [11]. α1-AT has multiple biological functions, of which its main function is to protect the lung against elastases produced by neutrophils [11,12,13,14]. However, it is also related to different pathological processes, such as cancer. The types of cancer with which it has been associated are breast, prostate, lung, cervical, bladder, and colorectal cancer, among others [12,15,16,17,18,19]. Also, all solid tumors induce a local or systemic inflammatory state, and α1-AT inhibits the enzymatic function of neutrophil elastase secreted by neutrophils during inflammation [20].

α1-AT is codified by the *SERPINA1* gene, located in the chromosome 14 locus (14q32.13) in humans. In addition, it consists of five exons and four introns [21,22], and it is composed of 418 amino acids with a molecular weight of 46.73 kDa [22]. Soluble levels of α1-AT can increase due to a wide variety of inflammatory processes, infections, cancer, liver disease, or pregnancy [21,23,24]. During the acute phase response, α1-AT levels increase by up to four times [23,25].

In Mexico, there are few studies in which circulating levels of the α1-AT protein have been evaluated, so the objective of this study was to evaluate the association of circulating levels of the α1-AT protein with the molecular subtype and progression in women with BC. Our results suggest that α1-AT could influence and favor an inflammatory state in BC, and its soluble levels increase in women with BC compared to healthy women (HW).

## 2. Materials and Methods

### 2.1. Patients

A total of 255 women with confirmed BC diagnosed histopathologically and 53 HW participated, and all participants had prior informed consent (IC) approved by the ethics committee from Centro de Investigación Biomédica de Occidente (CIBO) (CI-1305), and approval date December 2022. In addition, this study was performed according to the ethical principles for experiments involving humans stated in the Declaration of Helsinki, and participants were enrolled in this cross-sectional study in order to evaluate the association of soluble levels of α1-AT according to molecular subtype and clinical stage in women with BC.

### 2.2. Identification of Molecular Subtypes and Clinical Stage

The intrinsic molecular-like subtypes were identified by immunohistochemistry (IHC), according to the St. Gallen consensus 2013 recommendations (2015-updated): Luminal A-like, Luminal B-like, HER-2, and TN. Also, the TNM clinical stage classification system was used and separated into the I, II, III, and IV groups, according to TNM.

### 2.3. Quantification of Soluble Levels of α1-AT

Blood samples were obtained from women with BC and HW by venipuncture. The serum was collected following centrifugation for 5 min at 15,000 rpm and stored at first at a temperature of −20 °C, and then was placed in −80 °C until analysis. α1-AT soluble levels were measured by commercial ELISA kits (Invitrogen^®^ ThermoFisher, Waltham, MA, USA) Human Serpin A1 ELISA kit Cat. No. EH SERPINA1). The α1-AT assay limit detection was 2 ng/mL. The optical density was immediately determined, using a microplate reader set to 450 nm, according to the manufacturer’s instructions. The results were expressed as ng/mL.

### 2.4. Computational Analysis

#### Analysis of SERPINA1 mRNA Expression in Breast Cancer

To evaluate *SERPINA1* mRNA expression in BC, the GEPIA [26] (http://gepia.cancer-pku.cn, accessed on 18 October 2024) platform, which integrates data from normal samples obtained from TCGA and GTEx, was used.

The comparison of *SERPINA1* expression with clinicopathological features in BC was performed using cBioPortal [27] (https://www.cbioportal.org, accessed on 18 October 2024), specifically through the “Breast Invasive Carcinoma from TCGA—PanCancer Atlas” study.

GEPIA [26] and cBioPortal [27] are essential tools for querying and visualizing genomic data in cancer, facilitating the interpretation, analysis and graphical representation of genetic information in different types of cancer.

### 2.5. Statistical Analysis

The statistical analysis was performed using GraphPad Prism v5.0 software. The distribution of variables was analyzed with the Shapiro–Wilk normality test, and the variables with nonparametric distribution were expressed as a median. The Mann–Whitney U test was used to evaluate differences between two groups, and the Kruskal–Wallis test was used to analyze differences between three or more groups followed by Dunn’s test for multiple comparisons. In this study, the sample size was calculated using the Kelsey formula. All differences were considered statistically significant at *p* < 0.05.

For the statistical analysis of the computational analyses, a logarithmic scale was applied, using log2(TPM + 1). To determine significant differences in expression, cut-off criteria of Log2FC ≥ 1 and a *p* value ≤ 0.05 were established.

## 3. Results

In the present study, 255 women diagnosed with BC (mean 51 ± 13 years) and 53 HW were included. The demographic and clinical characteristics were analyzed; 61.6% of the women were in a postmenopausal status, and 92.5% of the women were classified in clinical stages (II, III, and IV) in concordance with Mexican stage distribution. With respect to the molecular subtype, Luminal A, Luminal B, and TN were predominant at 85.9% of the cases, distributed as follows: Luminal A with 36.5%, Luminal B with 25.1%, and TN with 24.3% (Table 1).

### 3.1. Soluble Levels of α1-AT in Women with BC and HW

**Figure 1:**
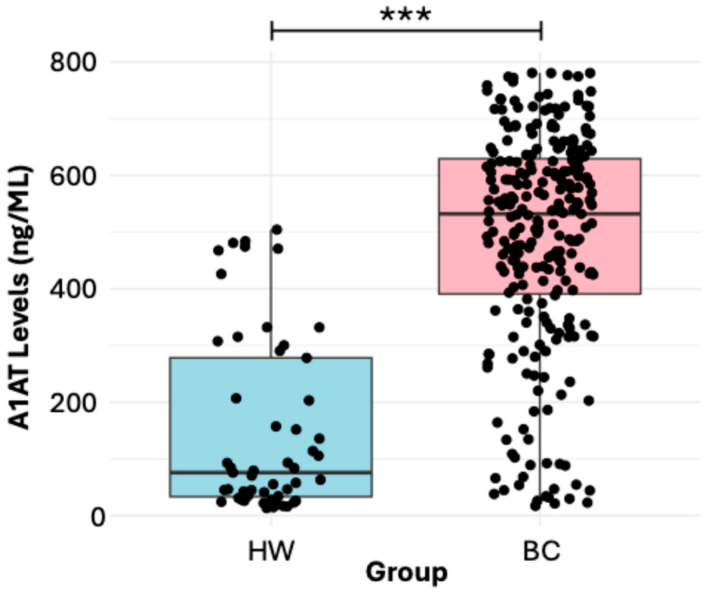
Soluble levels of α1-AT in HW and women with BC. Statistical analyses were performed using the Mann–Whitney U test; horizontal lines show the median. The analyzed groups HW vs. women with BC show significant differences, *p* value (*** *p* < 0.001).

**Figure 2:**
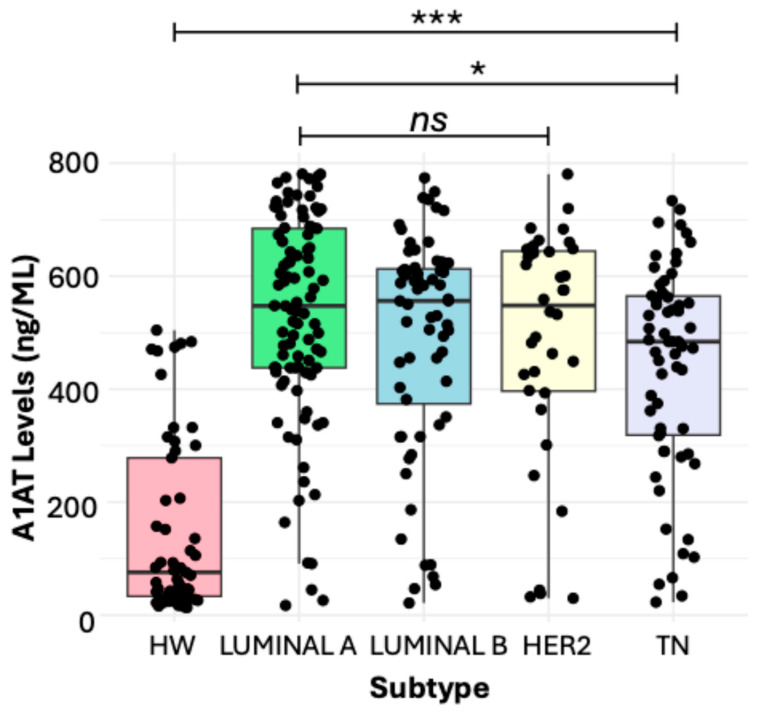
Soluble levels of α1-AT comparing HW vs. molecular subtypes. The comparison of HW vs. molecular subtypes showed significant differences, *** *p* < 0.001. When comparing soluble levels of α1-AT according to the molecular subtypes, the molecular subtype Luminal A vs. TN showed significant differences, *p* value (* *p* = 0.03), and finally, Luminal A, Luminal B, and HER2 did not show significant differences in soluble levels. Statistical analyses were performed using the Kruskal–Wallis test. Horizontal lines show the median. *ns*, not significant.

The soluble levels of α1-AT were significantly higher in women with BC (532.2 ng/mL) in comparison with those in HW (75.8 ng/mL), and we found significant differences between the groups (*p* ˂ 0.001) (Figure 1). A detailed analysis showed that the levels of α1-AT with respect to the molecular subtype showed significant differences between Luminal A (547.5 ng/mL) and TN (484.1 ng/mL) with a *p* value = 0.03. Luminal A, Luminal B, and HER2 did not show significant differences between groups (Figure 2). Additionally, we did not find significant differences between clinical stages I (515.4 ng/mL), II (505.5 ng/mL), III (538.2 ng/mL), or IV (544.0 ng/mL), only when comparing with HW (Figure 2). We also evaluated the relationship between the groups according to body mass index (BMI). In this study, the women with BC had higher BMIs with a percentage of 30.9% overweight and 44% obesity, while the normal weight represented 25.1%; we did not observe a significant association between the groups. Additionally, other clinical variables were also analyzed, including age at menopause (younger than 45 and older than 45) and Ki67 levels. However, they did not show significant differences in soluble levels of α1-AT between groups.

**Figure 3:**
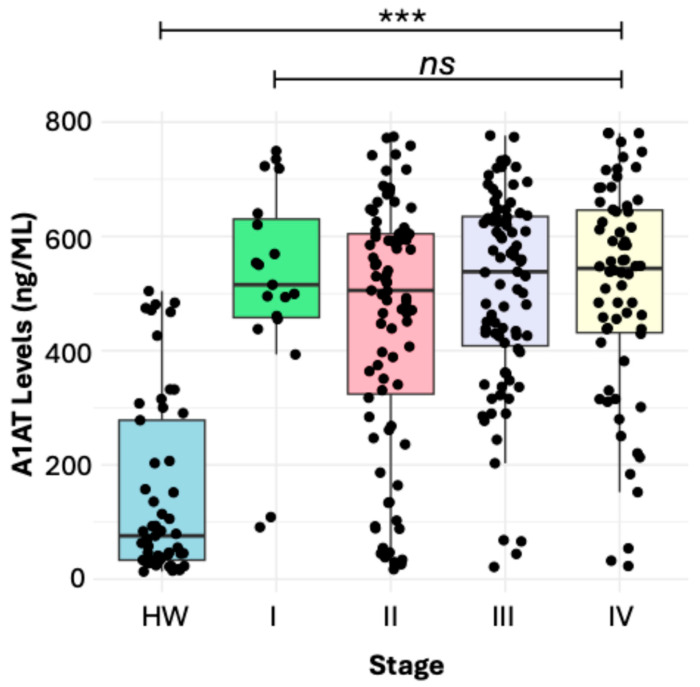
Soluble levels of α1-AT between HW and clinical stage. The data showed significant differences between HW in comparison with all clinical stages, *** *p* < 0.001. However, when comparing according to clinical stage, the data showed no significant differences between groups. Statistical analyses were performed using the Kruskal–Wallis test for three or more groups. The horizontal lines show the media. *ns*, not significant.

Furthermore, when α1-AT levels were compared in samples from HW, with all clinical stages of BC (I-IV), significant differences were observed between both groups. However, no differences were found in the soluble levels of α1-AT when compared to each other (*p* < 0.05) (Figure 3).

### 3.2. Overview of SERPINA1 in BC

In the “Breast Invasive Carcinoma of TCGA—PanCancer Atlas” study, *SERPINA1* was observed to have mutations in approximately 2% of the samples profiled for this gene (996 in total). Seven variants of uncertain significance (VUS) were identified, including four missense mutations, two truncations, and one splice variant (Table 2).

**Figure 4:**
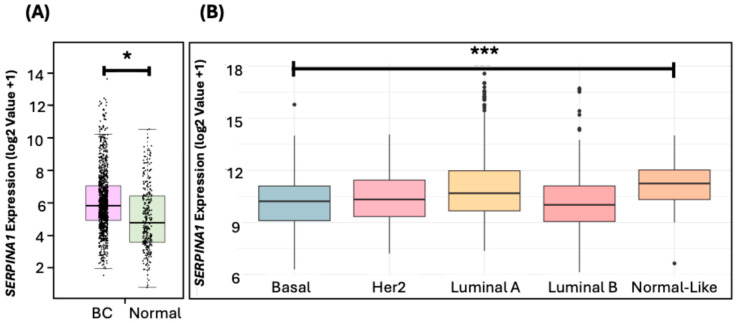
Expression of *SERPINA1* in BC versus normal tissue (**A**) and across different BC subtypes (**B**). * indicates *p* < 0.05, *** indicates *p* < 0.0001.

When comparing *SERPINA1* mRNA expression between BC and normal tissue, we observed higher expression in BC (5.83) compared to normal tissue (4.78) (* *p* < 0.05) (Figure 4A).

When *SERPINA1* expression was compared between BC subtypes (Normal-like, Luminal A, Luminal B, Her2, and Basal-like), significant differences were observed between them (χ^2^ = 47.017, *** *p* = 1.513 × 10^−9^). The Normal-like subtype presented the highest median *SERPINA1* expression with 11.53, followed by Luminal A (10.67), Her2 (10.30), Basal-like (10.24), and Luminal B (10), which showed the lowest expression (Figure 4B). In addition, when each of the subtypes was compared, significant differences were observed between several subtypes (Table 3), highlighting the differences between Basal-like and Luminal A, as well as between Basal-like and Normal-like, with variability in *SERPINA1* expression observed between these groups.

**Figure 5:**
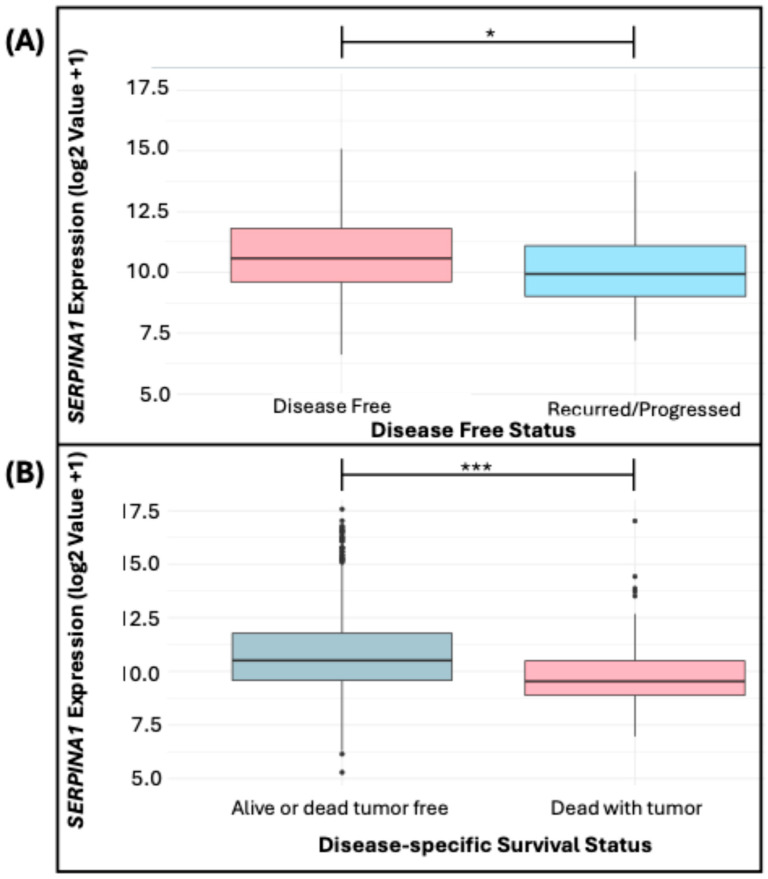
Expression of *SERPINA1* in “Disease Free” Status (**A**) and Disease-Specific Survival Status (**B**). * indicates *p* < 0.05, *** indicates *p* < 0.0001.

When comparing *SERPINA1* expression between the “Recurred/Progressed” and “Disease Free” patient groups, a statistically significant difference in the medians of gene expression was observed (* *p* = 0.0033). The median *SERPINA1* expression in “Disease Free” patients was 10.6, whereas in “Recurred/Progressed” patients, it was 9.94 (Figure 5A). In addition, the analysis revealed significant differences between the groups according to their survival status (*** *p* = 3.493 × 10^−5^). In the group “Alive or Dead Tumor Free”, the median *SERPINA1* expression was 10.50, in contrast to the “Dead With Tumor” group, which showed a significantly lower median of 9.53 (Figure 5B).

## 4. Discussion

The α1-AT protein has a wide range of biological functions, and its main function is to protect the lungs against elastases produced by neutrophils. However, it is also related to different pathological processes such as cancer. There is evidence that soluble levels of α1-AT are increased in different cancer types: prostate, lung, cervical, and breast, among others [11,13,19,28,29]. According to current studies, it has been demonstrated that high expressions of α1-AT relate to poor prognoses in different types of cancer, including BC [19,29,30]. In this study, we found a significant increase in the soluble levels of α1-AT in BC patients compared to HW (Figure 1), and this agrees with the results obtained in the evaluation of soluble levels of α1-AT in patients with other types of cancer, such as those with colorectal cancer [17] and lung cancer [29]. These increased levels in BC patients could be due to the process of inflammation that occurs in cancer patients [31] and the possible capacity of tumor cells to synthesize α1-AT with the purpose of promoting a microenvironment that helps cancer survival [11,28]. This relates to the results obtained in the evaluation of markers of epithelial-to-mesenchymal transition in lung cancer, where the authors found that the overexpression of α1-AT reduced the expression of E-cadherin and induced the expression of *N*-cadherin and Vimentin, which is associated with the migration and invasion of cancer cells [29].

Multiple factors are responsible for the disease’s onset, promotion, and progression [32,33]. In this instance, the imbalance of some molecules present in serum, such as interleukins and growth factors, has been associated with cancer [34,35,36], and other molecules have gained importance. For example, α1-AT is a protein that is synthesized mainly by hepatocytes, and although α1-AT deficiency has been associated with liver and lung disease [37,38,39], its participation in carcinogenic processes has been evaluated in recent years. It has been observed that the increase in serum concentrations of α1-AT is common in some malignant diseases such BC [40]. In the present study, statistically significant differences were observed in soluble levels of α1-AT between women with BC and clinically healthy women. These findings are compatible with those found by [15] in other types of cancer where they observed a significant increase in serum levels of α1-AT in patients with lung and prostate cancer when compared with those of the control group. However, no differences were observed in BC [15]. On the other hand, a case-control study conducted by [41] reported that more than 90% of patients with lung cancer had higher concentrations compared to those of the control group [41].

Regarding the molecular subtypes, no differences were observed in the soluble levels of α1-AT when the four subtypes were compared. However, there were differences in the serum concentrations of α1-AT in samples from patients with triple-negative BC (TN) with Luminal A. In the latter, a significant increase was observed compared to TN. The differences were likely due to tumor heterogeneity and the possible onset of metastasis to visceral organs such as the liver in patients with TN [42]. A study conducted by [43] evaluated the metastatic behavior of BC subtypes and concluded that this type of cancer was not associated with lower liver metastases [43]. Consequently, liver damage in patients with TN may contribute to a decrease in α1-AT concentrations, since the liver is the main organ responsible for its synthesis. On the other hand, experimental studies have shown that the activation of the estrogen receptor promotes the release of IL-33, capable of promoting airway inflammation in murine models. In this sense, it is known that α1-AT exerts an anti-inflammatory effect, and based on the findings previously described, it is possible that patients with estrogen-positive cancer subtypes also produce a higher concentrations of the protein in response to the inflammatory state [44].

Furthermore, other experimental studies have described that an increase in α1-AT promotes the development of metastasis in the lungs, although the molecular mechanisms are unknown [45]. It has been observed that Luminal A BC is a type of neoplasia that is characterized by developing metastatic lesions in non-visceral organs such as lung and bone [46], and it is possible that for this reason, the soluble concentrations of α1-AT are higher in this type of neoplasia to favor the development of metastatic disease in the lungs. In contrast, some studies have evaluated the expression of α1-AT in tumor tissue from patients with TN, and it was observed that the level of expression was higher compared to those in other molecular subtypes and healthy tissue; however, the sample size was not significant in that study [19]. Finally, immunohistochemical studies have described that Luminal BC subtypes present nuclear accumulation of the p53 protein, which indicates mutations in the gene [47]. In this regard, studies with lung cancer cell lines have shown that mutant p53 can drive oncogenic pathways that modulate the expression of some genes; among them, the gene that encodes the α1-AT protein was identified as a critical effect [48].

Although few studies have evaluated the participation of α1-AT with BC, it is possible that in the future it could be considered as a marker for prediction and tumor progression with clinical applications because our findings are compatible with those described in previous studies which have reported differences in the concentration of α1-AT between HW and patients with BC. For example, it has been observed that in clinical stages II–III in BC, there is a greater expression of α1-AT precursors compared to that in clinical stage I, and there are also reports that demonstrate a greater frequency in the expression of the protein in tumors with a larger size, classified in the T3–T4 category [49].

Other research on BC has described that the downregulation of α1-AT is associated with the invasion of axillary lymph nodes, possibly because a poor concentration of α1-AT is not sufficient to prevent the degradation of the extracellular matrix by proteases [50], which is an important phenomenon in the development of metastases [51]. Furthermore, it may be that the expression level of the protein under study is dependent on the activity of small miRNAs such as miR-214 because a decrease in the expression levels of α1-AT has been observed in tumor tissue from patients with TN after the upregulation of miR-214 [52]. In this sense, it is known that miRNAs are regulators of gene expression [53] and that there is the possibility that α1-AT, like other molecules such as nitric oxide, plays a dual role in the pathophysiology of BC [54,55]. However, more studies are necessary to expand the information and verify these hypotheses.

Regarding clinical stages, in our study, no differences were observed between soluble α1-AT concentrations and stage. This finding is different from what was described by [56] in other types of cancer, since they observed that in more advanced stages of intrahepatic cholangiocarcinoma, the expression levels of α1-AT were higher [56]. In fact, in bladder cancer, the detection of urinary α1-AT has been proposed as a possible marker in the detection of this type of neoplasia [57], and in colorectal cancer, it has been observed that the soluble concentrations of the protein increase as the clinical stage increases [58]. Furthermore, other studies have shown that in non-small-cell lung cancer (NSCLC), an increase in the serum concentration of α1-AT is a marker of poor prognosis [59], which could be associated with more aggressive stages and clinical signs of the disease. It is likely that in the present study, α1-AT concentrations were similar between the clinical stages of patients with BC because all participants were receiving cytotoxic chemotherapy prior to the time the samples were collected, and it is known that damaged liver disease is one of the most common adverse effects of cytotoxic treatment [60,61]. In this sense, hepatic cytotoxicity could trigger an imbalance in the enzymatic and functional status of the hepatocyte [62], which could alter the synthesis of α1-AT and mask the basal concentration that patients without prior cytotoxic treatment may be producing.

**Figure 6:**
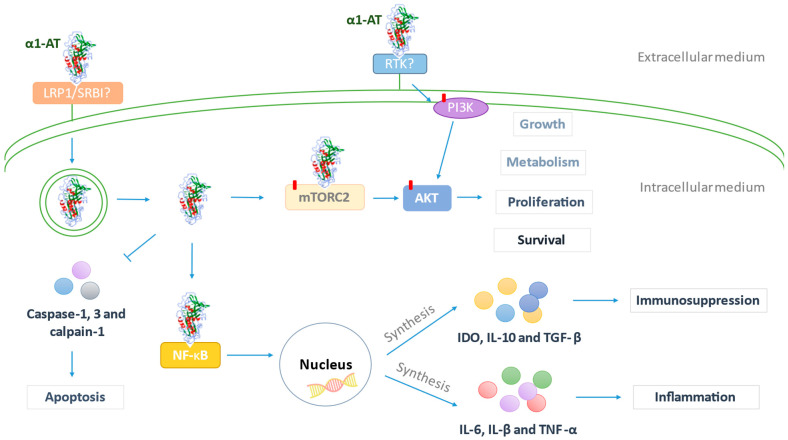
Mechanisms of action in cancer progression mediated by α1-AT. The protein internalization to cells due to the union to LRP1 (low-density lipoprotein receptor-related protein 1) and SR-BI (scavenger receptor class b type I), leading to the endocytosis of the protein by mechanisms that continue to be unknown. Subsequently, α1-AT is liberated in the cytoplasm and interacts with the mTORC2 complex, activating the intermediate in the PI3K/AKT/mTOR pathway, AKT (protein kinase B). The phosphorylation of this protein promotes growth in cells, metabolism, proliferation, and cell survival. However, PI3K/AKT/mTOR activation may also be mediated by the interaction between α1-AT and RTK (tyrosine kinase receptor), allowing for the activation of the protein PI3K (phosphatidylinositol 3-Kinase) and therefore, the phosphorylation of the intermediate in this pathway. Moreover, once α1-AT is internalized and exposed in the cytoplasm, it could interact with NF-kB (nuclear factor kB), inducing immunosuppression mediated by the nuclear genes that codify for IDO, IL-10, and TGF-β, as well as the synthesis of the pro-inflammatory cytokines L-6, IL-β, and TNF-α, which also possess the capacity to increase the levels of α1-AT. Finally, it may inhibit the apoptotic activity of caspase-1, 3, and calpain-1, promoting cancer survival.

In addition, in BC cells, it has been previously described that cancer survival could be favored by the ability of α1-AT to activate the PI3K/Akt/mTOR pathway, which develops fundamental functions such as inhibiting apoptosis [19] and promoting growth, metabolism and proliferation [63]. Also, it favors cell survival mediated by the inhibition of caspases in lung cells [64] and contributes to immunosuppression by stimulating the synthesis of IL-10, TGF-β, and idoleamine 2,3-dioxygenase (IDO) in dendritic cells in murine models [65]. Also, through the NFkB pathway, it favors the production of proinflammatory cytokines (Figure 6). Furthermore, in another study, researchers quantified the increased soluble levels of the proinflammatory cytokines IL-1β, IL-6, TNFα, and cytokines produced by the Th17 profile in women with BC compared to HW [66].

Additionally, it has been reported that high expressions of α1-AT are associated with advanced stages (III–IV) compared with early stages (I–II) in several types of cancer, including cervical [13], lung [29], and breast [19] cancer. More importantly, α1-AT levels decrease significantly after surgery [67] and chemotherapy [11], suggesting that α1-AT has potential as a diagnostic and therapeutic marker in various types of cancer. However, in the current study, we did not find significant differences between soluble levels of α1-AT and clinical stages.

Furthermore, a study conducted by [13] identified that serum biomarkers in cervical cancer patients included α1-AT, which exhibited significantly different expressions between serum samples from healthy control patients and patients with cervical cancer. In addition, Western blot analysis results indicated that patients with stage III and IV cancer had higher expression levels of α1-AT compared to stage I–II patients. However, in this study, we did find significant differences between the early and advanced stages. In another study, where the measurement of soluble protein levels was carried out before and after treatment, it was highly significant in lung and prostate cancer. The researchers found that associated soluble levels were highly significant in advanced stages. Also, prostate cancer demonstrated a direct and significant correlation between the elevated levels of the serum α1-AT and the stage of cancer [11].

Finally, the pathophysiological and molecular mechanisms of α1-AT in cancer are still unclear. However, α1-AT could be participating in different biological and molecular processes in the tumor microenvironment, which could cause an increase in systemic concentrations [68]. Likewise, the genetics of the Mexican population are the result of interbreeding between American native, European, Asian, and African subjects, and this intensive mix could explain the differences between populations. Our results could be compared with other populations, due to the mixed race that the Mexican population presents, as well as being part of different research. These results indicate the importance of geographic localization and ethnic origin even in the same country [69].

### Computational Analysis

In the “Breast Invasive Carcinoma of TCGA—PanCancer Atlas” study, mutations in *SERPINA1* were identified in approximately 2% of the samples analyzed, including variants of uncertain significance. Currently, the mutational status of *SERPINA1* in BC and other cancers is not fully elucidated. Therefore, an analysis investigating the role of specific mutations in this gene and their relationship to BC and other tumors is needed.

When comparing *SERPINA1* expressions between BC tissues and normal tissues, significantly higher expressions were observed in BC, suggesting that *SERPINA1* may play a role in the development or progression of BC. Previously, it was described that *SERPINA1* overexpression was also observed in cutaneous melanoma [70], pancreatic ductal adenocarcinoma [71], colorectal cancer [72], and non-small-cell lung cancer (NSCLC) [25]. In these contexts, this gene plays an active role in several cellular processes associated with cancer, such as inflammation, migration, and metastasis.

It was also observed that Normal-like and Luminal A subtypes had higher *SERPINA1* expressions, while Basal-like, HER2, and Luminal B subtypes exhibited lower levels. A previous study [73] suggested that high *SERPINA1* expressions may predict better clinical outcomes in ER+ and ER+/HER2+ patients, since *SERPINA1* is a direct target gene of the estrogen receptor, and its regulation may be mediated by estrogen signaling; thus, this gene may play a protective role in the tumor progression of BC. We observed this trend in our results, where we observed a higher expression of *SERPINA1* in disease-free patients compared to those who experienced recurrence. This pattern suggests that *SERPINA1* may be related to better disease prognosis and response to treatment. Also, lower *SERPINA1* levels were associated with worse prognosis and higher mortality, highlighting the need to further investigate its role in BC pathogenesis.

In contrast, the overexpression of this gene in tumor cells and stroma has been associated with poor prognosis [74], whereas in pancreatic ductal adenocarcinoma, it acts as an oncogene that is associated with poor prognosis and short survival [71].

These differences reflect the complexity of the tumor microenvironment. In BC, *SERPINA1* may play a protective role, possibly due to its function as a direct target of the estrogen receptor. In contrast, in other cancers, *SERPINA1* is associated with poor prognosis. This suggests that, although *SERPINA1* has the potential to be a valuable marker in BC, its role may vary according to the type of cancer.

## 5. Conclusions

Soluble levels of the α1-AT protein increased in women with BC compared to HW. Furthermore, in our results, the Luminal A molecular subtype presented the highest soluble levels, while TN showed the lowest levels of all other subtypes. Our results suggest that α1-AT could influence and favor an inflammatory state in BC. Furthermore, in the computational analysis, we observed that *SERPINA1*, the gene encoding α1-AT, was overexpressed in BC with differential expression between subtypes. While this overexpression appears to contribute to an inflammatory state in BC, it may also play a protective role, highlighting a complex relationship in the tumor microenvironment.

## Figures and Tables

**Table 1:** General characteristics of women with BC.

Variable	*n*	Percentage (%)
Mean age at diagnosis (years)	255	
Mean ± SD	51 ± 13	
Minimum	25	
Maximum	84	
Hormone status		
Pre-menopausal	98	38.4
Post-menopausal	157	61.6
Body Mass Index		
Normal weight	64	25.1
Overweight	79	30.9
Obesity	112	44.0
TNM clinical stage		
I	19	7.5
II	87	34.1
III	83	32.6
IV	66	25.8
Molecular subtype		
Luminal A	93	36.5
Luminal B	64	25.1
HER2	36	14.1
Triple Negative (Basal-like)	62	24.3

**Table 2:** *SERPINA1* Mutations Identified in BC.

Protein Change	Mutation Type	ClinVar	Subtype
Y184 *	Nonsense	Pathogenic/Likely pathogenic	Her2
*X355_splice*	Splice		Luminal B
*P2A*	Missense		Luminal A
*G344R*	Missense	Conflicting interpretations	Luminal A
*V326I*	Missense	Conflicting interpretations	Her2
*D280N*	Missense		Luminal A
Q33Rfs*47	FS del		Luminal A

*: Indicates a premature termination codon (stop codon), generating a truncated protein.

**Table 3:** Comparison of *SERPINA1* expression across BC subtypes.

Comparison	Adjusted *p*-Value *
Basal-like vs. Luminal A	3.012888 × 10^−6^
Her2 vs. Luminal A	1.046070 × 10^−2^
Basal-like vs. Normal-like	2.237449 × 10^−4^
Her2 vs. Normal-like	4.660713 × 10^−3^
Basal-like vs. Her2	1.902457 × 10^−1^
Basal-like vs. Luminal B	9.243745 × 10^−2^
Luminal A vs. Luminal B	5.467099 × 10^−5^
Luminal A vs. Normal-like	7.981116 × 10^−2^

* Dunn post hoc test.

## Data Availability

Data and materials are available in the article.
